# Beef Tea

**Published:** 1902-12-06

**Authors:** 


### Editor's Letter-Box.

[Our Correspondents are reminded that prolixity is a great bar to publication, and that brevity of style and conciseness of statement greatly facilitate early insertion.]

### BEEF TEA.

The Matron of the Cranbrook Workhouse Infirmary writes:—My attention has been drawn to a letter in The Hospital in which pre-eminence is claimed for Bovril over beef tea.

As both these have been used in this Infirmary, allow me to endorse the claim set forth in favour of Bovril.

Bovril is cheaper, cleaner, more easily and promptly prepared than beef tea. But if these were its only merits it would not be tolerated in this or other institutions for the use of the sick.

It has far higher claims. It is admitted by those well qualified to pronounce judgment that its dietetic properties are unassailable.

